# Medicinal Effects of Indian Springs Water on Typhoid Fever

**Published:** 1908-01

**Authors:** A. F. White

**Affiliations:** Flovilla, Ga.


### *MEDICONAL EFFECTS OF INDIAN SPRINGS WATER ON TYPHOID FEVER.

BY A. F. WHITE, M. D., FLOVILLA, GA.

The secretion of urine is one of the most important of the functions of the human body, as it is through this channel that the greater portions of the nitroginized waste of the tissue gets out of the system.

Waste or destruction of tissue is just as important in the

*Read before the Sixth District Medical Society. Macon, Ga.

animal economy as the supply of nutrition, and we find that the retention of this waste is more serious in its results than the want of nutrition.

We will find hereafter that the urine contains elements that are poisonous to the human body and when retained in the blood in sufficient quantity, they exert the same influence that would follow the absorption of a narcotic poison. Further than this, we have already noticed when considering the pathology of fever that the nitrogonized material which is converted into urea and uric acid may undergo such changes by the failure of the kidneys to remove it as well as set up a process of change in living blood which will finally result in its death.

The urine consists on an average of water one thousand parts; solids, twenty parts; the specific gravity averaging 1020. The proportions between the fluid and the solid portions of the urine varies greatly in different persons, and in the same person at different periods of the day.

Thus a man may today void forty ounces of urine of specific gravity of 1015, and tomorrow but twenty ounces specific gravity of 1035 and though the quantity of urine has varied one half, the amount of solids of the actual secretion is the same in both cases.

The amount of urine passed in twenty-four hours being determined and its average specific gravity ascertained, it is very easy to calculate the amount of solids in it. We are to suppose, however, that we have determined the amount of recreation as the specific gravity may be changed by the presence of foreign elements in it as sugar albumen mucus and the salts of lime, potash and soda.

The solids of the urine are composed of urea, uric acids, fixed salts, organic matter and volatile saline combinations, the amount excreted during twenty-four hours in a healthy man being: urea 270 grains, uric acid 76 grains, fixed salts 150 grains, organic matter and volatile saline constituents 106 grains, or a total of 608.8 grains. Thus you can see the great importance of the rapid elimination of all of these nitrogonized wastes from the human body. And on examination of the elements of Indian Spring water you can clearly see the great medicinal properties of the water. It is so light it is freely eliminated through both the skin and kidneys, carrying off these poisons from the human body.

The three emunctories, the skin, the kidneys and the bowels, are the channels or great source of all eliminations. Now after we have clearly shown the full constituents of the urine, I will give you this analysis of the Indian Spring water, that you may see the adaptability of this water in typhoid fever.

This to U. S. gallon:

| | |
|---|---|
| Potassium Bromide | .0113 |
| Sodium Iodide | .0009 |
| Sodium Phosphate | .0032 |
| Ammonium Chloride | .0072 |
| Liithium Bicarbonate | .1933 |
| Potassium Chloride | .1326 |
| Sodium Chloride | 1.3872 |
| Sodium Sulphate | 2.9582 |
| Magnesium Bicarbonate | .8375 |
| Calcicum Bicarbonate | 3.2842 |
| Sodium Bicarbonate | 1.9592 |
| Sodium Silicate | .5235 |
| Silicen Dioxide | 1.4150 |
| Iron Sequioxide | .0297 |
| Organic and Volatile | .8863 |
| Total Solids | 13.6291 |

Therefore, not only in typhoid, but in other fevers, is the water of superior value to other waters.

You take a man who has been on a jag, or alcohol poisoning, and give him as much of this water as he can drink every hour; it will not be long before his desire for whiskey has ceased. Also in most diseases of the urinary apparatus, in cystitis, acute or chronic, it is almost a specific. In all diseases where the organs are lined with a mucous membrane, you will find this water of superior value, and in chronic gonorrhea or gleet, it is panacea..

